# *Allium cepa* Bio Assay to Assess the Water and Sediment Cytogenotoxicity in a Tropical Stream Subjected to Multiple Point and Nonpoint Source Pollutants

**DOI:** 10.1155/2019/5420124

**Published:** 2019-03-03

**Authors:** W. M. Dimuthu Nilmini Wijeyaratne, L. G. Y. J. G. Wadasinghe

**Affiliations:** Department of Zoology and Environmental Management, University of Kelaniya, Kelaniya, Sri Lanka

## Abstract

The present study was conducted to assess the cytotoxicity of water and sediments of an industrial effluent receiving water body in the western province of Sri Lanka using* Allium cepa* bioassay. Six sampling sites (Site A: Urban; B: Industrial; C: Water intake for public water supply; D: Industrial; E: Agricultural; F: Reference) were selected from the study area. Ten replicate water and sediment samples were collected from each site, and physical and chemical parameters were measured using standard analytical methods. Cytotoxicity of water and sediment elutriates were measured using* Allium cepa* bioassay. Despite the significant spatial variations, the overall water and sediment quality parameters of the study sites were in accordance with the standard ambient environment parameters to sustain a healthy aquatic life. In the* A. cepa* bulbs exposed to water samples, significant root growth variations were not observed within 48 hours of exposure. However, significant root length variations were observed in* A. cepa* bulbs exposed to sediment elutriates within the 48-hour exposure and the percentage root growth inhibition increased with increase of exposure time. Similar trend was observed in mitotic activity indicating significantly lower mitotic indices (compared to that of the reference site) in* A. cepa* root tip cells exposed to sediment elutriates than those exposed to water samples. Further, the highest number of nuclear abnormalities was recorded from root tip cells of* A. cepa* exposed to water and sediment samples from sites B, C, and D. Therefore, it is of extreme importance to identify the composition and speciation of these cytogenotoxic compounds in the tropical climatic conditions and to propose possible clean-up or treatment solutions to overcome this environmental and public health risk associated problem.

## 1. Introduction

Point and nonpoint source pollution is a major environmental issue in many aquatic ecosystems as both organic and inorganic toxic chemicals can be added to the aquatic system by intentional or unintentional human activities [1]. These toxic chemicals can be adsorbed by particulate matter in the water column and deposited on sediments and are subjected to various transformation processes within sediments which causes the sediments to act as both a sink and source of contaminants to the overlying water column and biota [2–4].

Exposure to organic and inorganic chemicals over a long period of time can cause ecological health impairment of aquatic ecosystems causing considerable effects on aquatic biota including bioaccumulation of chemicals in organisms and biomagnification in higher trophic levels. Further, these can result in cytotoxic and genotoxic effects in the organisms [5, 6]. Therefore, many ecotoxicological studies focus on the assessment of physical and chemical environmental parameters and biological responses of organisms [7–11]. However, recent ecotoxicological studies are paying more attention in using bioassays to assess the mutagenic and genotoxic effects of aquatic pollution [6, 12, 13].

These mutagenic and genotoxic studies have focused on assessing genotoxicity and mutagenic effects of fish species [12, 14], microorganisms [15, 16], mammals [17, 18], and higher plants [19, 20] in relation to variation of chemical parameters in aquatic ecosystems. However, compared to other organisms* Allium cepa* is considered as an efficient bioindicator in genotoxicity testing, because of the rapid root growth rate and reduced number of large chromosomes [6, 21, 22].* A. cepa* assay is commonly utilized as a short-term and cost-effective indicator of toxicity in monitoring water pollution in many parts of the world. This bioassay can provide valuable information on the presence of genotoxic and/or mutagenic compounds in surface waters and sediments of aquatic ecosystems.* Allium cepa* bioassay can be used to assess cytotoxic and genotoxic endpoints such as chromosomal aberrations, nuclear alterations, root growth inhibition, and mitotic index alterations [23–27].

However, in Sri Lanka, very few studies have focused on assessing the cytotoxicity and genotoxicity of organisms in their natural environment [6, 28–30]. Even these conducted studies have focused on correlations between cytotoxic and genotoxic responses only in relation to water quality parameters of the aquatic system. However, as sediments can also influence cytotoxic and genotoxic response in organisms it is important to assess the effect of contaminated sediments on cytogenotoxic alterations of biota. Therefore, the present study aims to assess the cytotoxic effects of water and sediments of an industrial effluent receiving water body in the western province of Sri Lanka using* Allium cepa* bioassay. The screening would provide valuable information about the presence of genotoxic and/or mutagenic substances in surface waters and sediments by indicating the ability of such substances to induce chromosomal aberrations in* A. cepa* root cells.

## 2. Materials and Methods

### 2.1. Sampling Sites

**Figure 1:**
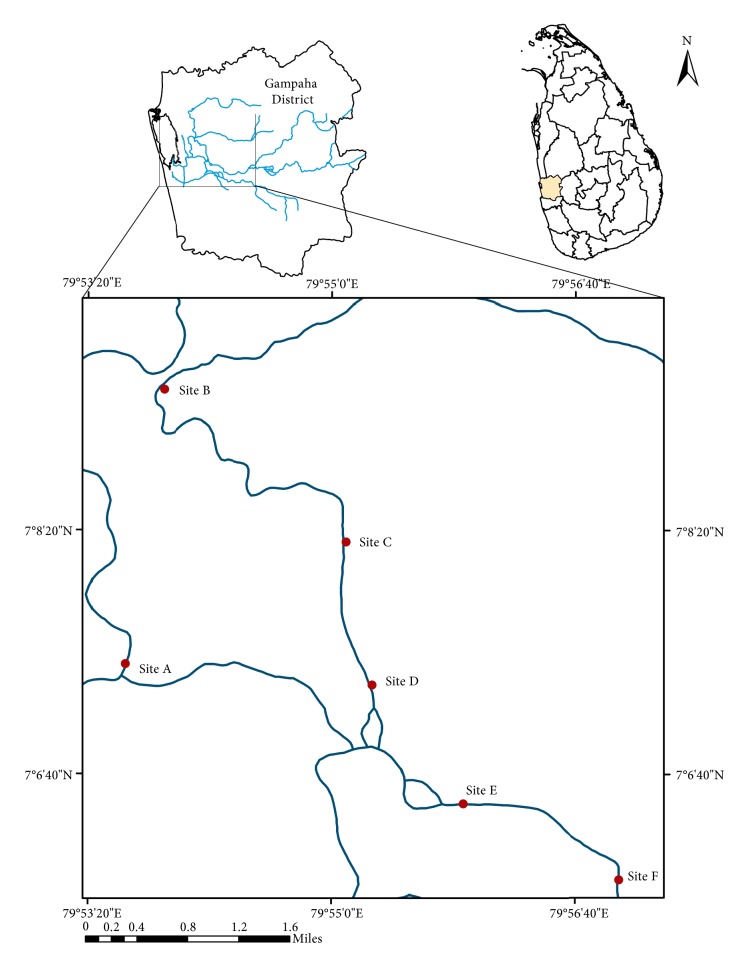
Map of the study area showing sampling sites (Site A: Urban; Site B: Industrial; Site C: Water intake for public water supply; Site D: Industrial; Site E: Agricultural; Site F: Reference site).

Dandugan oya (7°7′29.28′′N, 79°51′35.29′′E) is a stream located in the western province of Sri Lanka and is receiving industrial waste from multiple point and nonpoint sources. It also serves as a raw water source for public water supply in some suburban areas. Six sampling sites with different adjacent land use areas were selected for the present study. The location of sampling sites in the Dandugan oya is given in Figure 1. Site A (7°13′38.20′′N, 79°89′01.9′′E) was located near heavily populated urban area and was receiving urban waste water and sewer inputs. Sites B (7°07′45.5′′N 79°55′07.6′′E) and D (7°09′44.3′′N 79°53′40.0′′E) were located at heavily industrial regions and were receiving point source inputs of industrial discharges from multiple types of industries including tanneries, textile industries, and rubber processing industries. Site C (7°08′00.2′′N 79°55′03.4′′E) was the site of water intake for public water supply and was located in between the two industrial sites. Site E (7°06′50.7′′N 79°55′39.2′′E) was located in an area where extensive paddy cultivation is carried out. Site F (7°06′01.9′′N 79°57′05.6′′E), which was located further upstream from the other sites, was considered as the reference site as this site contained minimum anthropogenic interventions compared to other sites (Figure 1).

### 2.2. Analysis of Water and Sediment Quality Parameters

Surface water samples and shallow sediment samples (0-0.4m depth) were collected in 10 replicates from each site. Sampling was conducted at two month intervals from May to November 2017.

### 2.3. Analysis of Water Quality Parameters

At each sampling site, water pH, temperature, conductivity, total dissolved solids (TDS), and salinity were measured in situ using a calibrated digital multiparameter (YSI Environmental Model-556 MPS). Dissolved oxygen concentration (DO), biochemical oxygen demand 5 days after incubation (BOD_5_), chemical oxygen demand (COD), nitrate concentration, and phosphate concentration of water were analyzed following standard methodologies [31].

### 2.4. Analysis of Sediment Quality Parameters

At each sampling site, sediment pH and conductivity were measured in situ using the calibrated digital multiparameter (YSI Environmental Model-556 MPS). Sediment organic matter content was measured in the laboratory using the loss on ignition method and the percentages of sand, silt, and clay content of the sediments were measured using the sedimentation jar.

*Allium cepa Test.* Commercial variety of common onion (*Allium cepa*) was used for the determination of different toxicity end points of meristematic cells. Equal sized healthy onion bulbs were chosen and the outer scales of bulbs were removed by gently scraping to make the apices of root primodia exposed. Scarped onion bulbs were germinated in glass test tubes containing distilled water for 24 hours in the dark. The* Allium cepa* bioassay in accordance with Grant (1982) [32] with some modifications was conducted using water samples collected from the study sites. After 24 hours onion bulbs were exposed to the exposure media (70 mL, composite sample taken from each site) in the glass tubes at the time of processing. For each exposure media 10 onion bulbs were tested. Bulbs with exposure media were kept in dark to avoid the direct sunlight.

For sediment toxicity testing, the* Allium cepa* bioassay was conducted using sediment elutriates. Sediments were thawed, weighted (wet weight), and combined with distilled water to make sediment: water (1: 4 ratio recommended by Daniels et al., 1989[5]) slurry. Solution wad was stirred manually for 5 minutes. Then solution was followed by centrifugation for 5 minutes at 1500 X G. Supernatant was decanted and stored at 4°C for sediment bioassay. Scarped onion bulbs were germinated in glass tubes containing distilled water for 24 hours in the dark. After 24 hours onion bulbs were exposed to the exposure media (70 mL, prepared elutriate mixture) in the glass tubes at the time of processing. For each exposure media, 10 onion bulbs were tested. Bulbs in exposure media were kept in dark to avoid the direct sunlight.

After 48 hours of exposure, root lengths of randomly selected five onion bulbs from each exposure media were measured in millimeters. Root tips (5-6 from each onion bulb) of 1-2 mm length were processed for microscopic studies of toxicity end points. Root tips were fixed in ethanol: glacial acetic acid (3:1, v/v) solution for overnight at 4°C. Root tips were transferred into 70% alcohol and stored at 4°C until the time of processing.

When processing the root tips, they were hydrolyzed in hydrochloric acid (1N) solution for 5 minutes at 60°C and washed with distilled water. Root tips were then placed in watch glasses containing acetocarmine for 30 minutes to allow the stain to penetrate to the primordial cells. After staining, root tips were placed on glass slides and a slight pressure was applied on the cover slip to squash the tip cells over the slide. Prepared slides for each exposure medium was observed under the light microscope at 400x magnification to score of mitotic stages, occurrence of micronuclei, and nuclear abnormalities in the interphase cells.

Mitotic index was calculated as the number of dividing meristematic cells in 100 total meristematic cells by counting 1000 meristematic cells in each slide [33, 34].

(1) Mitotic  index=Number  of  dividing  meristemetic  cells1000  total  meristemetic  cells×100

Nuclear abnormalities were detected in 1000 observed interphase cells. The frequency of nuclear abnormalities including micronuclei was detected in 1000 observed interphase cells and recorded. Remaining bulbs were exposed to the media continuously followed by daily renewal of exposure media. After seven days root lengths of the bulb were measured in millimeter to assess the phytotoxicity end points.

### 2.5. Statistical Analysis

After confirming for normality, using Anderson Darling test, the spatial variation of physical and chemical parameters of water and sediments were analyzed using one way ANOVA followed by Tukey's pairwise comparison. The percentages of sand, silt, clay, and TOC were arcsine transformed before analysis. One way ANOVA followed by Tukey's pairwise comparison was used to examine the differences in tested parameters relevant to the onion bulbs exposed to the water and sediment elutriates of the study sites. Accepted level of significance was p < 0.05. MINITAB 14 software was used for statistical analysis of data.

## 3. Results

### 3.1. Spatial Variation of Water Quality Parameters

Spatial variation of water quality parameters in the study sites is given in Table 1. There was no significant spatial variations in water pH, temperature, nitrate concentration, total phosphate concentration, BOD_5,_ and salinity (Table 1, P>0.05). Significantly lower dissolved oxygen concentration was recorded from sites C and D (ANOVA, Tukey's Pairwise comparison, p<0.05). Further, significantly high chemical oxygen demand was also recorded from Site D (Table 1, ANOVA, Tukey's Pairwise comparison, p<0.05). Significantly high total dissolved solids and conductivity were recorded from sites A and B (Table 1, ANOVA, Tukey's pairwise comparison, p<0.05).

### 3.2. Spatial Variation of Sediment Quality Parameters

Spatial variation of sediment quality parameters in the study sites is given in Table 2. There was no significant spatial variations in percentage of total organic matter and sediment pH (Table 2, ANOVA, Tukey's pairwise comparison, p<0.05). Sites B, C, and D showed significantly lower percentage sand content and sites B and C recorded significantly higher percentage silt content (Table 2, ANOVA, Tukey's pairwise comparison, p<0.05). Site A recorded significantly lower percentage clay content and Site D recorded significantly higher percentage clay content compared to other sites (Table 2, ANOVA, Tukey's pairwise comparison, p<0.05). Sites E and F recorded significantly lower sediment conductivity and sites A, B, and D recorded significantly higher sediment conductivity compared to other sites (Table 2, ANOVA, Tukey's Pairwise comparison, p<0.05).

### 3.3. Root Growth Pattern of* Allium cepa* Bulbs

The spatial variation of mean ± standard deviation (SD) of root lengths and percentage root growth inhibition of* A. cepa* bulbs exposed to water samples collected from study sites is given in Table 3. The root lengths of* A. cepa* bulbs after 48-hour exposure to water samples ranged from 0.058 cm to 5.772 cm and after 7-day exposure ranged from 0.064 cm to 5.772 cm. There was no significant spatial variation in the root lengths after 48-hour exposure to water samples. However, after 48-hour exposure, the percentage root growth inhibition compared to the reference site varied as Site A(23.9%)>B(19.2%)>E(16.7%)>D(7.5%)>C(2.1%) (Table 3). After 7-day exposure, the* A. cepa* bulbs exposed to water from sites B and C showed significantly lower root length compared to other sites (Table 3, ANOVA, Tukey's pairwise comparison, P<0.05) and the percentage root growth inhibition compared to the reference site varied as Site B(43.9%)>C(38.1%)>E(18%)>D(17.4%)>A(11.8%) (Table 3).

The spatial variation of mean ± standard deviation (SD) of root lengths and percentage root growth inhibition of* A. cepa* bulbs exposed to sediment elutriates collected from study sites is given in Table 4. The root lengths of* A. cepa* bulbs after 48-hour exposure to sediment elutriates ranged from 0.000 cm to 2.572 cm and after 7-day exposure to sediment elutriates ranged from 1. 52 cm to 5.26 cm. After 48-hour exposure, sites B and C showed significantly lower root length compared to the reference site (Table 4, ANOVA, Tukey's pairwise comparison, P<0.05) and the percentage root growth inhibition compared to the reference site varied as Site C(29.7%)>B(25.8%)>E(12.4%)>A(12.2%)>D(12.0%) (Table 4). After 7-day exposure to sediment elutriates, a similar trend was observed in the root lengths and the percentage root growth inhibition. However, higher percentage root growth inhibition was observed in all sites after 7-day exposure compared to 48-hour exposure (Table 4).

### 3.4. Toxicity Assessment by* A. cepa* Bio Assay

**Figure 2:**
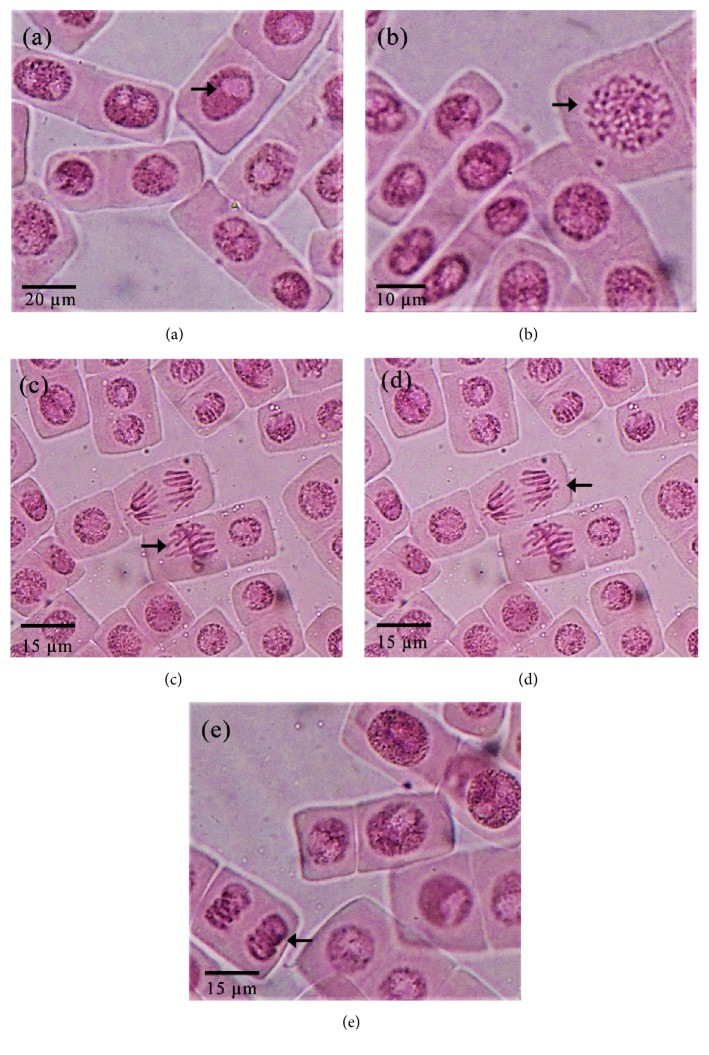
The microscopic appearance of normal interphase cells and dividing cells in the* Allium. cepa* root tip meristematic region ((a): Interphase; (b): Prophase; (c): Metaphase; (d): Anaphase; (e): Telophase).

**Figure 3:**
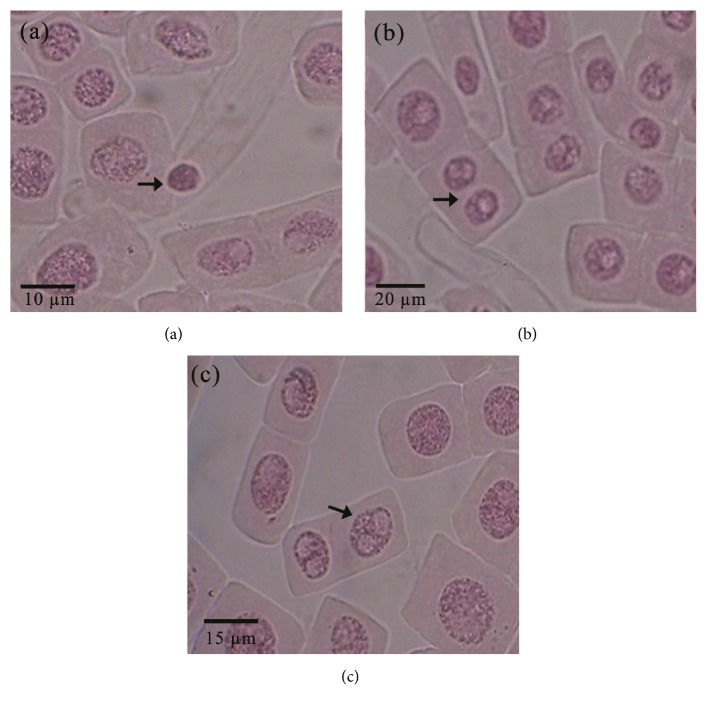
The microscopic appearance of the observed nuclear abnormalities in the* Allium cepa* rot tip cells ((a): condensed nuclei; (b): bi nuclei; (c): nuclear buds).

The microscopic appearance of normal interphase cells and dividing cells in the* A. cepa* root tip meristematic region is given in Figure 2 and the microscopic appearance of the observed nuclear abnormalities is given in Figure 3. Nuclear abnormalities observed in the dividing cells of onion root tips were nuclear buds, bi nuclei, and condensed nuclei (Figure 3).

**Figure 4:**
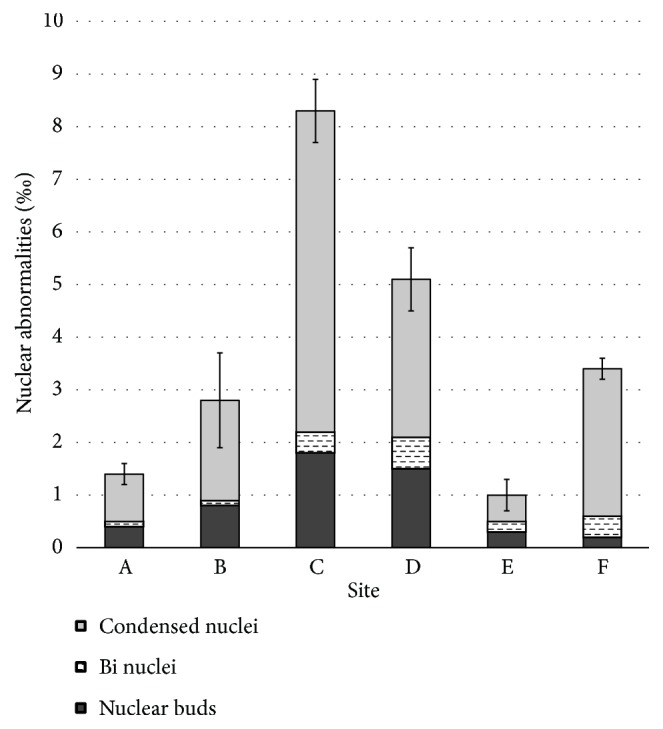
The percentage occurrence of nuclear abnormalities observed in the dividing cells of* Allium cepa* root tips exposed to water samples collected from the study sites in Dandugan oya.

The percentage occurrences of nuclear abnormalities observed in the dividing cells of onion root tips exposed to water samples collected from the study sites in Dandugan oya are given in Figure 4. The dividing cells of onion root tips exposed to water samples of the water intake site for the public water supply (Site C) showed significantly higher total nuclear abnormalities (8.3%) compared to other sites (Figure 4). The dominant form of nuclear abnormality in all the sites was condensed nuclei while the number of binuclei recorded was comparatively lower than that of the other abnormalities. The dividing cells of onion root tips exposed to water samples from Sites A (1.4%) and E (0.9%) showed significantly lower total nuclear abnormalities compared to other sites (Figure 4). All the observed nuclear abnormalities were significantly high in the dividing cells of onion root tips exposed to water samples from Site C. In addition to Site C, the dividing cells of onion root tips exposed to water samples from sites B (0.8%) and D (1.5%) showed significantly higher nuclear buds formation compared to other sites. The highest percentage of binuclei formation was observed in the dividing cells of onion root tips exposed to water samples from Site D (0.6%) (Figure 4).

**Figure 5:**
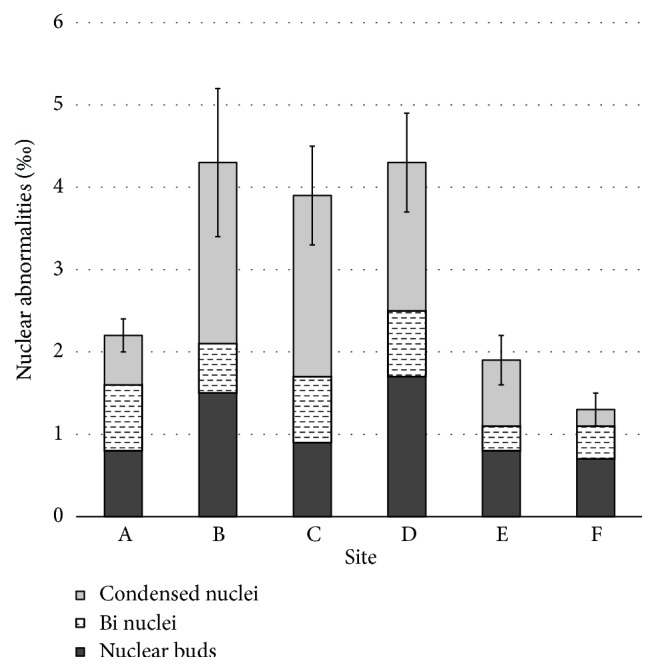
The percentage occurrence of nuclear abnormalities observed in the dividing cells of* Allium cepa* root tips exposed to sediment elutriate samples collected from the study sites in Dandugan oya.

The percentage occurrence of nuclear abnormalities observed in the dividing cells of onion root tips exposed to elutriates of sediment samples collected from the study sites in Dandugan oya is given in Figure 5. The dividing cells of onion root tips exposed to sediment elutriates from Sites B (4.2%), C (3.9%), and D (4.4%) showed significantly higher total nuclear abnormalities compared to other sites (Figure 5). Significantly high occurrence of nuclear buds were observed in the dividing cells of onion root tips exposed to sediment elutriates from sites B (1.5%) and D (1.7%). Significantly high occurrence of binuclei was observed in the dividing cells of onion root tips exposed to sediment elutriates from sites A (0.8%), C (0.8%), and D (0.8%). Significantly high occurrence of condensed nuclei was observed in the dividing cells of onion root tips exposed to sediment elutriates from sites B (2.2%), C (2.2%), and D (1.8%) (Figure 5).

Mean± standard deviation of the mitotic index of root tip cells of* Allium cepa* bulbs following exposure to water and sediment elutriates collected from study sites is given in Table 5. Mitotic index of the root tip cells of* A. cepa* bulbs exposed to water samples ranged from 1.5% to 6.3%. Sites C and D showed significantly lower mitotic index values compared to other sites. The root tip cells of* A. cepa* bulbs exposed to water collected from the reference site (Site F) showed the highest mean mitotic index (6.0%) compared to other sites and the lowest mitotic index was recorded from the root tip cells of* A. cepa* bulbs exposed to water collected from the water intake site for the public water supply (Site C) (1.8%). The differences were significant at 95 % level of significance (ANOVA, Tukey's pairwise comparison) (Table 5).

Mitotic index of the root tip cells of* A. cepa* bulbs exposed to sediment elutriate samples ranged from 2.5% to 7.6%. The root tip cells of* A. cepa* bulbs exposed to sediment elutriates collected from the reference site (Site F) showed the highest mean mitotic index (7.4%) compared to other sites and the lowest mitotic index was recorded from the root tip cells of* A. cepa* bulbs exposed to sediment elutriates collected from the water intake site for the public water supply (Site C) (2.9%). The differences were significant at 95% level of significance (ANOVA, Tukey's pairwise comparison) (Table 5).

## 4. Discussion

Dandugan oya is subjected to pollution due to numerous nonpoint source pollutants including industrial, agricultural, and urban pollutant inputs. According to the proposed ambient water quality standards published by the Central Environmental Authority of Sri Lanka, in inland waters of Sri Lanka, the total nitrate concentration should be ≤ 5 mg/L, total phosphorous (TP) concentration should be ≤ 0.4 mg/L, dissolved oxygen content should be ≥ 3 mg/L, BOD_5_ should be ≤ 5 mg/L, and pH should be within 6-8.5 in order to maintain healthy aquatic life [35]. Therefore, the water quality parameters of the study sites of this study were in accordance with the standard ambient water quality parameters to sustain a healthy aquatic life. However, the results of the bioassay indicated that possibility of occurrence of cytogenotoxicity in the water and sediments of Dandugan oya.* Allium cepa* bio assay is a popular technique in assessing the cytotoxicity and genotoxicity in the aquatic environments due to its cellular proliferation kinetics, the rapid root growth rate, large numbers of cells in division, easy management, and less number of large chromosomes [6, 33, 34, 36]. In the present study, the root growth inhibition of the* A. cepa* bulbs exposed to water and sediment samples collected from study sites were compared with that of the reference site. The results indicated a root growth inhibition in all sites compared to the reference site. However, the percentage root growth inhibition trends in the* A. cepa* bulbs exposed to water samples were different from that of those exposed to sediment elutriates. In the* A. cepa* bulbs exposed to water samples, significant root growth variations were not observed within the first 48 hours of exposure and significant changes of root growth were observed after 7 days of exposure. However, significant root length variations were observed in the* A. cepa* bulbs exposed to sediment elutriates within the first 48-hour exposure and the percentage root growth inhibition of all the sites, except Site D, increased with increase of exposure time (Table 4). A similar trend was observed with the mitotic activity as well, indicating significantly lower mitotic indices (compared to that of the reference site) in the* A. cepa* root tip cells exposed to sediment elutriates of study sites than those exposed to water samples (Table 5). The significant reductions of root length and mitotic activity are considered as indicators of rhizotoxicity, being a general phenomenon caused by most pollutants [37, 38]. In the aquatic environment, sediments play a key role as they provide the basis of aquatic ecosystems by providing a substrate for many aquatic biota and being a deposition substrate for many suspended and dissolved matter. The results of the present study indicate higher significant reductions in root length and mitotic activity of* A. cepa* bulbs exposed to sediment elutriates compared to water samples indicating high phytotoxicity in sediments. Therefore, these results indicate that the sediments of Dandugan oya are acting as storage of cytotoxic and genotoxic compounds to overlying water column and biota. Sediments in the stream ecosystems are characterized by presence of fine particles and many aquatic pollutants are predominantly associated with fine deposits that are rich in organic matter [39]. In the present study, the highest root growth inhibition and lowest mitotic index were recorded from the* A. cepa* bulbs exposed to sediment elutriates from sites B and C and these sites had significantly high percentage silt content compared to other sites. The high percentage silt content in these two sites may be facilitating the binding of toxic compounds and chemicals to the sediments and thereby resulting in high rhizotoxicity in* A. cepa* bulbs.

Further, the mitotic index of the* A. cepa* root tip cells of the present study ranged from 1.5 % to 7.6 %. A mitotic index less than 22% is recorded to be lethal to the organism [26]. Therefore, the mitotic indices recorded in the present study can be considered in the lethal range and may indicate high cytotoxic effects in the environment.

The occurrence of nuclear abnormalities in* A. cepa* root tip cells indicates the possibility of occurrence of genotoxic compounds in the exposed medium [6, 40]. In the present study, the highest number of nuclear abnormalities was recorded from the root tip cells of* A. cepa* exposed to the water and sediment samples collected from sites B, C, and D. Sites B and D were located in heavily industrial sites and field observations indicated that these sites were receiving industrial waste water from multiple point and nonpoint sources. These industrial wastes may be rich with cytotoxic and genotoxic compounds including heavy metals, poly aromatic hydrocarbons, and other organic and inorganic compounds that can trigger cellular and genetic variations in biota in these waste water receiving areas of the stream. Studies have proven that compounds such as polyaromatic hydrocarbons, copper, arsenic, and other industrial effluents can display observable cytotoxic and genotoxic effects in the* A. cepa* root tip cells [6, 25, 39–41]. Site C is the water intake site for the public water supply and the onion root tip cells exposed to water and sediments from this site exhibited significantly high nuclear abnormalities compared to other sites. Site C is located in between the industrial sites (Sites B and D), which also exhibits significantly high nuclear abnormalities. As this site serves as the water intake site for public water supply for suburban regions of this area, it is very important to consider pretreatment of water to destroy cytotoxic and genotoxic compounds before distributing water to general public in order to overcome public health risks.

## 5. Conclusions

The results of the present study indicated the potential occurrence of cytogenotoxic compounds in the water and sediments of the Dandugan oya, which is a tropical stream receiving multiple pollutants from point and nonpoint sources. As this stream also serves as a water intake site for public water supply of this area, it is of extreme importance to identify the composition and speciation of these cytogenotoxic compounds in the tropical climatic conditions and to propose possible cleanup or treatment solutions to overcome this environmental and public health risk associated problem. Further, continuous monitoring and management of water quality are of extreme importance to maintain the ecological health of the ecosystem.

## Figures and Tables

**Table 1:** Spatial variation of water quality parameters in sampling sites during the study period. Data are presented as mean ± standard deviation (SD). Results indicated by different superscript letters in each row are significantly different from each other (ANOVA, Tukey's test, p < 0.05, n=10).

	site A	site B	site C	site D	site E	site F
Water pH	6.89 ± 0.1^a^	6.42 ± 0.1^b^	6.15 ± 0.1^bc^	6.17 ± 0.1^bc^	6.00 ± 0.1^c^	5.94 ± 0.1^c^
Water Conductivity (*µ*S/cm)	4457 ± 833^a^	1630 ± 309^b^	90.60 ± 3.34^c^	75.71 ± 1.37^c^	68.94 ± 2.38^c^	70.61 ± 1.99^c^
Temperature (°C)	31.07 ± 0.4^a^	31.98 ± 0.2^a^	32.06 ± 0.2^a^	31.92 ± 0.3^a^	29.44 ± 0.2^b^	29.52 ± 0.1^b^
Water TDS (mg/L)	2375 ± 453^a^	740 ± 144^b^	44.58 ± 1.81^b^	35.73 ± 0.59^b^	32.01 ± 1.12^b^	32.97 ± 0.94^b^
Water DO (mg/L)	7.17 ± 0.71^a^	6.34 ± 0.65^a^	4.00 ± 0.34^b^	4.28 ± 0.41^b^	5.54 ± 0.13^ab^	6.19 ± 0.08^a^
Water Nitrate (mg/L)	0.55 ± 0.09^a^	0.39 ± 0.06^a^	0.43 ± 0.06^a^	0.40 ± 0.06^a^	0.32 ± 0.07^a^	0.37 ± 0.07^a^
Water Phosphate (mg/L)	0.044 ± 0.006^b^	0.082 ± 0.013^a^	0.052 ± 0.007^ab^	0.050 ± 0.008^ab^	0.038 ± 0.004^b^	0.043 ± 0.008^b^
Water BOD_5_ (mg/L)	1.44 ± 0.17^a^	1.30 ± 0.19^a^	1.18 ± 0.14^a^	1.87 ± 0.26^a^	1.77 ± 0.34^a^	1.28 ± 0.19^a^
Water COD (mg/L)	111.0 ± 10.8^b^	71.0 ± 9.8^b^	81.8 ± 7.4^b^	267.1 ± 56.5^a^	66.2 ± 11.8^b^	62.0 ± 11.3^b^
Water Salinity (^0^/_00_)	2.44 ± 0.469^a^	0.86 ± 0.170^b^	0.04 ± 0.001^bc^	0.04 ± 0.001^bc^	0.03 ± 0.001^c^	0.03 ± 0.001^c^

**Table 2:** Spatial variation of sediment quality parameters in sampling sites during the study period. Data are presented as mean ± standard deviation (SD). Results indicated by different superscript letters in each row are significantly different from each other (ANOVA, Tukey's test, p < 0.05, n=10).

	site A	site B	site C	site D	site E	site F
Sediment sand %	50.7 ± 4.5^a^	37.0 ± 1.3^b^	34.6 ± 1.9^b^	32.4 ± 2.5^b^	51.1 ± 3.1^a^	60.8 ± 3.1^a^
Sediment silt %	38.6 ± 5.0^a^	44.0 ± 4.0^b^	46.4 ± 2.0^b^	26.9 ± 3.9^c^	24.0 ± 2.5^c^	21.7 ± 3.0^c^
Sediment clay %	10.7 ± 2.2^a^	20.5 ± 2.4^b^	19.0 ± 1.7^b^	40.6 ± 4.9^c^	28.2 ± 2.2^d^	22.3 ± 1.8^d^
Shallow sediment TOM%	2.32 ± 0.24^a^	2.04 ± 0.11^ab^	1.61± 0.17^abc^	1.32 ± 0.12^bc^	1.88 ± 0.27^abc^	1.11 ± 0.18^c^
Shallow sediment pH	5.56 ± 0.30^ab^	5.86 ± 0.22^a^	5.85 ± 0.27^a^	5.62 ± 0.27^ab^	5.11 ± 0.09^ab^	4.71 ± 0.07^b^
Shallow sediment conductivity (*µ*S/cm)	5570 ± 727^a^	4206 ± 641^a^	535.7 ± 80.6^b^	195.3 ± 28.1^b^	36.36 ± 4.03^b^	25.29 ± 2.12^b^

**Table 3:** The spatial variation of root lengths and percentage root growth inhibition of *A. cepa* bulbs exposed to water samples collected from study sites. Data are presented as mean ± SD. Results indicated by different superscript letters in each column are significantly different from each other (ANOVA, Tukey's test, p < 0.05, n=5).

Site	48-Hour exposure		7-Day exposure	
	Mean root length	% growth inhibition compared to reference site	Mean root length	% growth inhibition compared to reference site
A	1.644 ± 0.177a	23.9	4.086 ± 0.210^a^	11.8
B	1.743 ± 0.100^a^	19.2	2.602 ± 0.134^b^	43.9
C	2.113 ± 0.120^a^	2.1	2.868 ± 0.188^b^	38.1
D	1.996 ± 0.113^a^	7.5	3.829 ± 0.206^c^	17.4
E	1.792 ± 0.117^a^	16.7	3.799 ± 0.240^c^	18.0
F	2.159 ± 0.128^a^	-	4.635 ± 0.199^a^	-

**Table 4:** The spatial variation of root lengths and percentage root growth inhibition of *A. cepa* bulbs exposed to sediment elutriate samples collected from study sites. Data are presented as mean ± SD. Results indicated by different superscript letters in each column are significantly different from each other (ANOVA, Tukey's test, p < 0.05, n=5).

Site	48-Hour exposure		7-Day exposure	
	Mean root length	% growth inhibition compared to reference site	Mean root length	% growth inhibition compared to reference site
A	2.158 ± 0.129^b^	12.2	4.037 ± 0.206^a^	13.4
B	1.823 ± 0.179^a^	25.8	2.600 ± 0.188^c^	44.2
C	1.728 ± 0.101^a^	29.7	1.875 ± 0.153^c^	59.8
D	2.162 ± 0.102^b^	12.0	4.022 ± 0.117^a^	13.7
E	2.184 ± 0.111^b^	12.4	3.830 ± 0.203^b^	17.9
F	2.458 ± 0.336^b^		4.664 ± 0.192^a^	

**Table 5:** Mean mitotic index of root tip cells of *Allium cepa* bulbs following exposure to water and sediment elutriates collected from study sites. Data are represented as mean ± SD. Results indicated by different superscript letters in each column are significantly different from each other (ANOVA, Tukey's test, p < 0.05, n=10).

Site	Mitotic index (%)	
	Water	Sediment elutriate
A	5.4 ± 0.6^a^	4.2 ± 1.4^a^
B	5.4 ± 1.0^a^	6.1 ± 0.5^c^
C	1.8 ± 0.5^b^	2.9 ± 0.5^b^
D	3.1 ± 0.3^c^	3.4 ± 0.6^a^
E	5.2 ± 0.9^a^	3.4 ± 0.7^a^
F	6.0 ± 0.6^a^	7.4 ± 1.1^c^

## Data Availability

The raw data of the study can be made available upon request.
